# Expression patterns of miR-21 and miR-146 in ulcerative colitis: implications for disease progression and therapeutic strategies^☆^

**DOI:** 10.1016/j.jgeb.2026.100770

**Published:** 2026-07-22

**Authors:** Bilal Rmaidh Mohammed, Huda Rafaa Sabbar AL-alwani, Raghad Jawad Hussein A.L. Akayshee

**Affiliations:** a Department of Microbiology, College of Medicine, University of Anbar, Al-Anbar Governorate, Ramadi, Iraq; b Department of Microbiology, College of Medicine, University of Anbar, Al-Anbar Governorate, Ramadi, Iraq; c Gastrointestinal and Hepatology Teaching Hospital, Al-Baghdad Governorate, Baghdad, Iraq

**Keywords:** Ulcerative colitis, microRNA, miR-21, miR-146, qRT-PCR, biomarkers and inflammation

## Abstract

**Background:**

Ulcerative colitis (UC) is a chronic inflammatory bowel disease (IBD), where dysregulated microRNAs (miRNAs) may play a role in the immune imbalance and injury to the mucosa. miR-21 and miR-146 have been associated with inflammatory signaling pathways.

**Objective:**

The aim of this study was to evaluate the expression levels of miR-21 and miR-146 in blood and colonic tissue of patients newly diagnosed with Ulcerative Colitis (UC) compared to healthy controls.

**Methods:**

A case–control study was performed from January to October 2025. Patients who have recently been diagnosed with UC. The age range of the study subjects was 15–65 years and they were recruited at the time of diagnosis from gastroenterology/endoscopy services inxx and from collaborating clinics and healthy individuals as controls. Total RNA extracted from peripheral blood and colonic tissue, and expression of miR-21 and miR-146 levels were quantified by qRT-PCR, Relative miRNA expression levels were calculated by the 2^-ΔCt method after normalization with the endogenous reference gene. The relative expression levels was calculated to the control groups.

**Results:**

Expression of miR-21 and miR-146 in blood and tissue was significantly lower in patients than in the controls.

**Conclusions:**

The new findings were that miR-21 and miR-146 were down-regulated in blood and much more strongly suppressed in colonic tissue in newly diagnosed UC. The results further confirm miR-21 and miR-146 as potential biomarker candidates for UC characterization, and as potential starting points for miRNA-based therapeutic approaches. These patterns should be confirmed in larger cohorts of patients and correlated to disease severity, extent and treatment response using formal inferential statistics in future studies.

## 1 Introduction

Ulcerative colitis (UC) is a chronic inflammatory disease characterized by continuous involvement of the colon and rectum with mucosal inflammation.^1^ It is a chronic disease with episodes of remission and flare-up which can result in symptoms such as abdominal pain, diarrhea, rectal bleeding and weight loss.^2^ Although some of these have similar features, they can be differentiated based on genetic predisposition, risk factors, clinical, endoscopic and histological features. The exact cause of inflammatory bowel disease is still unknown.^3^ There is variation in the part of the colon involved. There are some patients with inflammation confined to the rectum (ulcerative proctitis), and some with more proximal disease. The term pancolitis is used for ulcerative colitis that involves the whole of the colon.^4^

MicroRNAs (miRNAs) are small non-coding RNAs approximately 21–24 nucleotides (nt) in length, which are able to regulate gene expression at the post-transcriptional level.^5^ miRNA influences various physiological processes including the regulation of cell cycle and homeostasis, cell survival, cell differentiation, cell expansion and apoptosis. Some of the miRNAs also regulate gut epithelial cell differentiation.^6^

The sequences containing miRNAs are found in intronic or exonic parts of non-coding genes, in intronic parts of protein-coding genes, and in intergenic regions.^7^

Most miRNAs are transcribed from DNA sequences to produce primary miRNAs (pri-miRNAs) which are processed to precursor miRNAs (pre-miRNAs) and mature miRNAs. Most miRNAs bind to 3′ UTR of the target mRNAs and negatively regulate the expression.^8^ As miRNAs bind to the prime untranslated regions (3′ UTR and 5′ UTR) and silence genes at the post-transcriptional level, they control a broad spectrum of cellular processes such as proliferation, differentiation, development, metabolism and apoptosis.^9^

MicroRNAs are emerging as significant regulators involved in the pathogenesis of UC. They affect epithelial barrier integrity, innate/adaptive immune activation, and inflammation-associated tissue remodeling, and are intriguing potential biomarkers and therapeutic targets.^10^ One of the key components of UC is disruption to the lining of the bowel. Dysregulated miRNAs can directly change tight junctions, permeability, apoptosis and mucosal healing.^11^

## 2 Patients and methods

### 2.1 Patients & study design

This was a case–control study conducted during the months of January to October 2025. Subjects were selected from the gastroenterology/endoscopy services and collaborating clinics, as well as from the private clinic, for the newly diagnosed UC patients aged 15–65 years, and from apparently healthy individuals for the healthy controls. Two hundred samples were taken from patients. One hundred from blood and the remaining one hundred samples were tissue biopsies. A total of two hundred samples were collected from the controls. There were 100 blood samples and 100 tissue samples of biopsy. It can be used to compare the expression of these molecular markers between these two groups, and provide statistically significant differences in miRNA expression patterns of miRNA in ulcerative colitis. The study was carried out in the Microbiology Department.

### 2.2 Sample of study

#### 2.2.1 Blood sample

Three milliliters of blood will be taken in EDTA containing sterile tubes, the blood will be sent to the laboratory and plasma will be separated and kept in the freezer at - 80°C until use. All the blood samples in EDTA were aliquoted into 200 μL from plasma , mixed with Trizol (200 μL,600 μL) (Invitrogen (USA)) for molecular study. The distribution of the study samples were as follows: The blood samples were taken from all infected patients and healthy individuals.

#### 2.2.2 Tissue biopsy sample

A small tissue fragment was retrieved by the endoscope and was promptly placed in TRIzol reagent for detection for miRNA expression analysis. Patients and control had the biopsy taken.

### 2.3 Molecular investigation

#### 2.3.1 Isolation of RNA using TRIzol™

For each patient and healthy individuals 200 μl of blood was placed into 1.5 ml tube containing 600 μl of Trizol and inverted many times for mixing. Thirty minutes incubation to permit complete dissociation of the nucleoproteins complex. The chloroform that was used for lysis (0.15 mL of TRIzol™ Reagent). Incubation for 20 minutes. Spin at 12,000 × g for 15 minutes. The mixture was separated into a lower red phenol-chloroform, and interphase, and a colorless upper aqueous phase. The aqueous phase (with RNA) was transferred to a clean tube. Precipitation of the RNA from the aqueous phase was done by addition of 0.45ml isopropanol. The mixture was allowed to incubate for 20 minutes. The samples were then centrifuged for 10 minutes at 12,000 × g. The total RNA precipitate is a whitish, gel-like pellet at the bottom of the tube. The supernatant was aspirated off with the micropipette. The pellet gets resuspended by 0.75 mL of 75% ethanol. Then the vortex was used to dissolve the pellet and centrifuge for 5 minutes at 7500 × g. Using micropipettor, supernatant was removed. To dry the RNA pellet the tube opened for 15 minutes. Then the pellet resuspended by 50 ul of RNase-Free water and incubated at 60C for 15 minutes by using thermomixer. All Total RNA samples were kept at -20 °C until ready for downstream application.

#### 2.3.2 RT-qPCR protocol

This procedure was divided into two phases , the first one is done through synthesis of cDNA from RNA through EasyScript® First-Strand cDNA Synthesis SuperMix. The following procedure was carried out:

Five microliters of each extracted total RNA were placed in new PCR tube. EasyScript reaction mix containing dNTPs, buffer and other necessary components which are added to each sample as 10ul. The MuLV Enzyme (reverse transcriptase) was then added to the reaction at a rate of 1ul/sample. One microliter random primer for (U6 snRNA) also 1 ul specific RT primer for (miR-146 ),^12^ and the volume completed up to 20ul by adding nuclease-free water. The second part of this protocol was performed by selecting the cDNA samples from patient and control at one PCR run; there were two PCR tubes, both patient and control, for each gene ( miR-146),( miR-21) and (U6 snRNA) used as the housekeeping gene in this study. The intensity of SYBR Green fluorescence was used for quantification. The reaction mix composed from component with their quantity as mentioned in table below:

The total volume of each qPCR reaction was 20 μl. The Universal qPCR Master Mix was used to provide the required DNA polymerase, dNTPs, MgCl₂ and reaction buffer. To achieve specific amplification of the target sequence, forward and reverse primers were added (1μL each; 10μM). The template DNA (5μL) was then added as the DNA to be amplified, and the reaction volume was adjusted using 3μl of nuclease free water.

To remove the bubbles, the solution was spun to collect the liquid in PCR tubes, which was done quickly (1 minute at 2000g) and the program Real-Time PCR was setup with indicated thermocycling protocol. The PCR cycle for all types miRNA of followed the same steps, starting with five minutes at 95 ° denaturation for one minute at 94 ° C to denature the DNA. After C, samples were extended for 1 min at 63 °C followed by annealing for 1 min at 72 °C. A final extension phase was performed after 35 cycles of denaturation, annealing and extension.

#### 2.3.3 Stem-loop reverse transcription (RT) and PCR primers for quantitative analysis of miR-21, miR-146a and U6 snRNA by reverse transcription (RT)-qPCR

The stem-loop reverse transcription and quantitative real-time PCR (RT-qPCR) assays were used to quantify the expression levels of miR-21 and miR-146a. Target-specific forward primers and reverse primers were used for amplification, and specific stem-loop RT primers were used for cDNA synthesis. U6 small nuclear RNA (U6 snRNA) was used as endogenous reference gene to normalize the data. All the primer sequences used in this study are listed in Table.^1^

### 2.4 Statistical analysis: descriptive statistics

Summarize demographic and clinical characteristics of UC patients and controls using means, standard deviations, medians, and interquartile ranges for continuous variables, and frequencies and percentages for categorical variables. Test for normality of continuous variables using Shapiro-Wilk test.

## 3 Results

### 3.1 The distribution of the patients and controls by gender

**Fig. 1:**
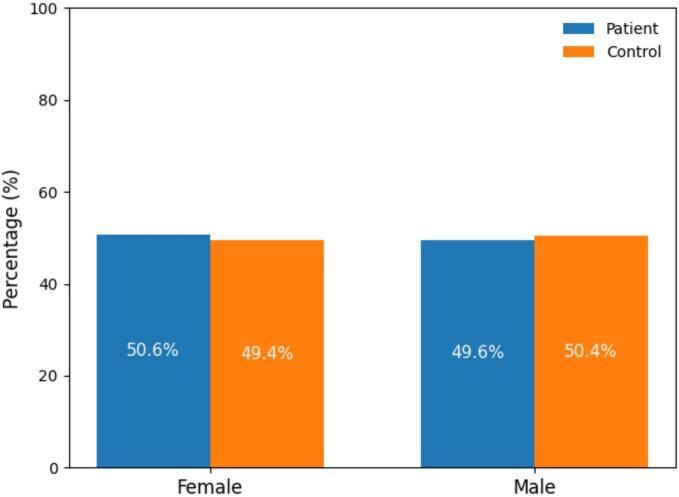
The distribution of patients and controls according to genders.

This study involved 200 participants, 100 with ulcerative colitis (UC) and 100 healthy controls. The study groups included 87 (43.5%) female and 113 (56.5%) were male. Of the females, 44 (50.6%) were UC patients, and 43 (49.4%) were controls groups, while 56 (49.6%) and 57 (50.4%) of the males were UC patients and control groups respectively. The distribution of gender was not significantly different between the UC patients and controls (p > 0.05), suggesting that gender matching was achieved between the two study groups. (Fig. 1). (See Table 1.)

**Table 1:** Stem-loop reverse transcription (RT) and PCR primers for quantitative analysis of miR-21, miR-146a and U6 snRNA by RT-qPCR.

Target	Primer Type	Sequence (5'→3')	References
miR-21	RT	GTCGTATCCAGTGCAGGGTCCGAGGTATTCGCACTGGATACGACTCAACA	^13^
miR-21	F	CGGCTAGCTTATCAGACTGA	^13^
miR-21	R	GTGCAGGGTCCGAGGT	^13^
miR-146	RT	GTCGTATCCAGTGCGTGTCGTGGAGTCGGCAATTGCACTGGATACGACAACCCA	^14^
miR-146a	F	GGGTGAGAACTGAATTCCA	^14^
miR-146a	R	CAGTGCGTGTCGTGGAGT	^14^
U6 snRNA RT	RT	CTCAACTGGTGTCGTGGAGTCGGCAATTCAGTTGAGAAAAATATG	^15^
U6 snRNA F	F	CTCGCTTCGGCAGCACA	^15^
U6 snRNA R	R	AACGCTTCACGAATTTGCGT	^15^

All primers were commercially synthesized by Macrogen Inc. (Seoul, South Korea)

Abbreviations: RT, reverse transcription primer; F, forward primer; R, reverse primer; miR, microRNA; U6 snRNA, U6 small nuclear RNA. Mature miRNAs were synthesized from RNA by using stem-loop RT primers and quantitative amplification was performed using gene-specific forward and reverse primers. The expression level of the miRNAs was normalized with respect to that of U6 snRNA. The 2^−ΔCt method was used to determine relative expression.

### 3.2 Expression of miRNA-21 in patients and controls

Various clinical specimens were used to showed expression of miRNA 21 of patients and controls. This work showed a reduction in the expression of miRNA-21 in the patients as compared to the controls in blood and tissue samples by 0.6 and 0.72 times, respectively (Table 2).

**Table 2:** Expression of miRNA-21 in patients and controls.

Sample	Group	Ct miRNA-21	Ct U6	Δ Ct	2^- Δ Ct^	Fold
Blood	Patients	33.445	24.066	9.38	0.001502	0.60
	Controls	27.036	18.406	8.63	0.002524	1.00
Tissue	Patients	23.9	16.8572	7.04	0.007584	0.72
	Controls	24.9978	18.4306	6.57	0.010546	1.00

### 3.3 Expression of miRNA-146 in patients and controls

Similarly, the expression of miRNA-146 was decreased in the blood sample of patients as compared to that of the controls by 0.67 and by 0.005 in the tissue sample, respectively (Table 3).

**Table 3:** Expression of miRNA-146 in patients and controls.

Sample	Group	Ct miRNA-146	Ct U6	Δ Ct	2^- Δ Ct^	Fold
Blood	Patients	42.449	25.026	17.423	5.6906E-06	0.67
	Controls	35.537	18.699	16.838	8.536E-06	1.00
Tissue	Patients	32.826	17.0585	15.7675	1.79271E-05	0.005
	Controls	25.6181	17.5564	8.0617	0.003742713	1.00

### 3.4 Comparison of the relative expression levels in miRNA-21 and miRNA-146 according to sample type

**Fig. 2:**
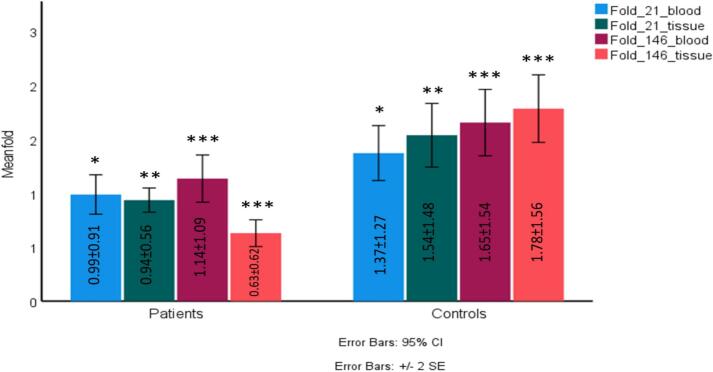
Relative expression of miR-21 and miR-146 in the blood and tissue of the ulcerative colitis patients and healthy controls. The expression levels were derived by using 2-ΔCt method and expressed as mean ± 95% CI.

**Fig. 3:**
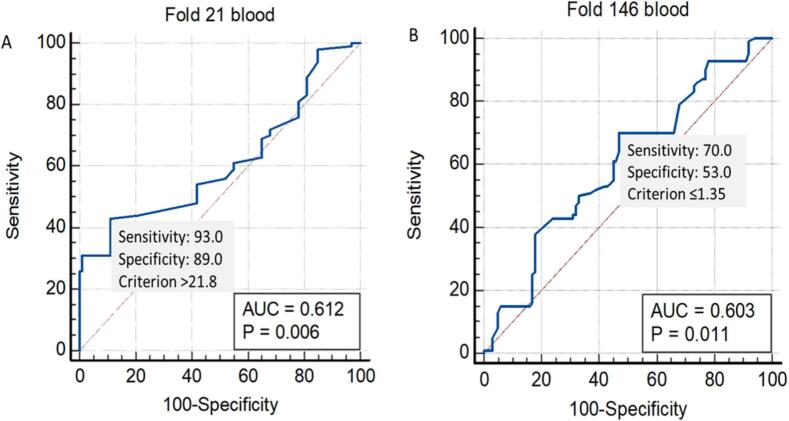
The ROC curve analysis of the miRNA-21 and miRNA-146 in blood samples.

**Fig. 4:**
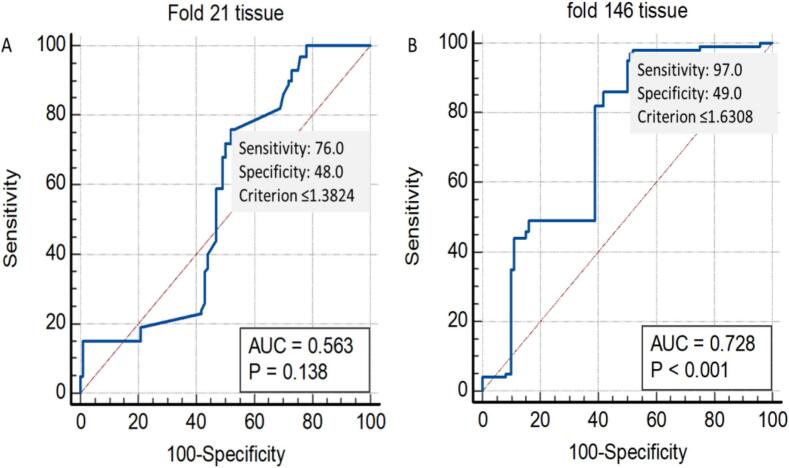
The ROC curve analysis of the miRNA-21 and miRNA-146 in tissue samples.

This study showed that patients had significantly lower levels of miR-21 and miR-146 than controls in both blood and tissue sample. The miRNA-21 expression was found to be reduced in the blood of patients (0.99) compared with controls (1.37), and statistically significant (p = 0.015). Likewise, miRNA-146 was significantly reduced in patients (Relative expression = 1.14) as compared to controls (Relative expression = 1.65; p = 0.007). These were more marked in tissue samples: There was a significant difference between patients (0.94) and controls (1.54; p ≤ 0.001) for miRNA-21 expression, and for miRNA-146 there was a strong difference between patients (0.63) and controls (1.78, p ≤ 0.001). Overall, two markers are both shown to be consistently and significantly downregulated in patients, with greater effects seen in tissue than blood. (Fig. 2). (See Fig. 3, Fig. 4.)

### 3.5 Receiver operating characteristic (ROC) curve analysis of variables investigated

A receiver operating characteristic (ROC) curve analysis was used to assess the diagnostic ability of the biomarkers studied to distinguish patients with ulcerative colitis (UC) from controls. As shown in Figure^3^ the area under the curve (AUC), optimal cut–off values, sensitivity, specificity, accuracy and statistical significance was determined for each variable.

### 3.6 Circulating miRNAs (blood samples)

Limited diagnostic performance was found for MiR-21 (blood) with AUC of 0.612 (p = 0.006). Sensitivity was low (93%) but specificity was relatively high (89%) at a cut-off ≤0.55.Although the modest AUC is statistically significant, the discriminative power of the test is poor to fair and therefore limits the use of the test in isolation in the clinical setting.

Likewise, miR-146 (blood) had an AUC of 0.603 (p = 0.011), and showed weak discriminatory power. It had moderate sensitivity (70%) and low specificity (53%) at a cut-off ≤1.35 with poor overall accuracy (23%). It is statistically significant but may not have a significant diagnostic value when used alone.

### 3.7 Tissue miRNAs

No statistically discriminative ability was found for MiR-21 (tissue) with an AUC of 0.563 (p = 0.138). The sensitivity (76%) and specificity (48%) were suboptimal, and the absence of statistical significance indicates that tissue miRNA-21 is not a good diagnostic marker of UC in this cohort. In contrast, MiR-146 (tissue) showed moderate diagnostic accuracy , with an AUC of 0.728 (p ≤ 0.001). At a cut–off value of ≤ 1.6308 it had very high sensitivity (97%) and low specificity (49%). This profile has potential use as a sensitive screening marker; however, its false-positive rate appears to restrict its specificity-based use as a diagnostic marker.

## 4 Discussion

The cause of ulcerative colitis is unknown, but there have been major advances in the understanding of the disease's pathogenesis. The cause is also not completely understood. Although colonoscopy combined with histopathological examination of the colon is the gold standard technique for the diagnosis of UC, it is invasive and has several limitations and hence it is necessary to search for some novel biomarkers for early diagnosis of the disease and treatment effect.^16^

The disease burden and prevalence of ulcerative colitis (UC, one of the types of IBD) is mentioned in the World Health Organization (WHO) report that UC is one of the incurable diseases of modern era with increasing prevalence. The progression to cancer in UC depends heavily on the extent, duration and intensity of the inflammation of the colonic mucosa.^17^

MiR-21 is an atypical miRNA that is expressed highly in many tissues/cells. The expression level of miR-21 is dysregulated in many diseases including cancer, infection and inflammatory diseases. The first estimation of the expression levels and its association with the risk of IBD was the previous meta-analysis of miR-21 to date.^18^

MiR-21 was also previously reported to be above the levels of non-inflammatory controls in patients with UC.^19^ But there are conflicting findings on the involvement of miR-21 in inflammatory processes. In inflammatory diseases, a few evidence has claimed that miR-21 expression might be decreased and might be anti-inflammatory; on the other hand, some studies have shown that miR-21 expression is increased.^18^

Other recent studies have investigated the implication of miRNAs in maintaining barrier function of the epithelia, e.g., miR-21 and miR-200B. Wild type and miR KO mouse models of DSS colitis in the murine have both increased survival, reduced tissue inflammation from wild types3 and this microRNA is known to target (Ras homolog family member B) Rhob which perturbs permeability of the gut.^20^

The results indicate that miR-21 and miR-126 may be used as potential biomarkers for active UC. Indeed, miR-21 may play a role in Ulcerative Colitis pathways, repressing Phosphatase and TENsin (PTEN) that led to the repressive activity of PTEN, and up-regulated PI3K/Akt activity, while contributing to the down-regulation of Programmed cell death protein 4 (PDCD4), Ras homolog family B (RhoB) and Nitric oxide synthase-2 (NOS2)-induced cellular damage.^21^

However, only a few studies have shown that there is upregulation of miRNAs in UC mucosa relative to controls. Indeed, miR-21, miR-29a, miR-126 and let-7f were found to be significantly increased in active UC patients compared to healthy controls, as demonstrated by Wu et al. By examining colonic mucosa from UC patients and control..^12^ The function and the target molecules of these isoforms are similar, and they also differ.^22^

MiRNA-146a is also involved in innate immune tolerance in newborns' intestine by protecting intestinal epithelial cells from being induced to apoptosis by bacteria. These studies suggest that miRNA-146a is induced by TLR signaling, but its main function could be to exert a dominant negative feedback effect to prevent uncontrolled inflammatory reactions after prolonged exposure to bacterial agents.^23^

MiR-146b was significantly up-regulated at day 3 post-DSS in parallel with diminished mucosal ulcerations and prolonged proliferation of PCNA + epithelial cells. This was opposed to the miR-146 down-regulation presented here. In the present study, the authors hypothesized that miR-146b could be used as an activator in the process of healing during IBD.^24^

Exposure to LPS, peptidoglycan and flagellin via TLR ligands also induces expression of miR-146a. It is important to be noted that mir-146a is NFκB-dependent miR which silences the downstream target genes of TLR signaling pathway like tumor necrosis factor (TNF)-receptor associated factor-6 and interleukin (IL)-1 receptor (ILR) associated kinase 1 controlling inflammation.^25^

Schaefer et al. 2015, found that three mucosal/bodily compartments (saliva, blood and stool) have distinct miRNA signature profiles in the spectrum of IBD. They also saw higher miR-146a expression in IBD patient intestinal biopsies than normal control patients and lower miR-146a gene expression levels in the blood samples of IBD patients compared to healthy controls.^26^

Clinical studies revealed that in blood and tissue the expression of miR-146a was lower in UC than in controls, and the expression of this miRNA was negatively correlated with the clinical and endoscopic activity of UC. In a second cohort of 312 UC patients, miR-146a was significantly lower in mild/moderate and severe disease compared to controls and maximal reduction was observed in the severe group. Moreover, the expression of miR-146a in the UC patients was inversely associated with pro-inflammatory mediators (NF-κB, CRP and IL-6 as well as TNF-a) during acute inflammation.^27^

Several reasons can be given as to why the concentration of microRNA is higher in tissues than in blood. Intracellular miRNAs are extremely abundant and play an important role in the regulation of genes, and typically, tissue assays reflect the presence of miRNAs in various cell types (tumor cells, stroma , immune cells, endothelium). The extracellular compartment (EV-encapsulated, protein-bound, lipoprotein-bound) is small, and is much more dilute than the intracellular compartment; this is detected by blood assays (plasma/serum). In the plasma, miRNAs are often mentioned to be in a "fairly low concentration", in comparison with that in cells/tissues^19^ .

Many miRNAs have tissue- or cell-type enrichment (e.g., epithelium, brain, liver, muscle). Thus, the tissue measurements correspond to high local transcription/biogenesis and high intracellular miRNA concentration, and the blood measurements correspond to a “system-level” signal that is mixed and diluted. Comparisons between tissue and circulating miRNAs highlight that the two compartments are related, but not identical, and that biological and release/clearance dynamics may constrain the correlation of these two compartments.^28^

Free miRNA is unstable in blood unless protected by: Extracellular vesicles (EVs),Argonaute2 (Ago2) complexes and HDL/lipoproteins .This protection explains why circulating miRNAs exist at all, but it also means the measurable pool is constrained to the protected fraction.^29^

Even if miRNAs are released into the blood, they can be cleared by incorporation in receiving cells, filtration/clearance (e.g. liver/spleen macrophage systems, kidney pathways depending on carrier) or degradation of unprotected molecules. Therefore, tissue over-expression doesn't necessarily translate to high blood levels: blood levels are determined by the rate of release and the rate of clearance. These delivery/uptake dynamics have been highlighted by reviews of the extracellular miRNA biology.^30^

The specimen (serum vs plasma) used is crucial for measured “blood miRNA” as coagulation and platelet activation may influence the measured miRNAs. Certain miRNA signals may be dominated by blood cells (RBCs, platelets, leukocytes) which also can introduce distortion, particularly in the case of hemolysis.^31^

The downregulation of miR-146a in UC is also clinically relevant as it has been linked to disease severity and as a potential biomarker of inflammatory activity. There was a significant correlation between miR-146a expression and disease activity index score, and endoscopic findings were more severe in patients with low miR-146a; this indicates possible use of miR-146a as a stratification marker of disease phenotypes and a response marker for therapeutic interventions.^32^

While the widely-used U6 snRNA was chosen here as an endogenous reference gene, the use of U6 snRNA for circulating miRNAs has been discussed. It has been reported that there is variation in the expression of U6 in samples derived from blood and caution should be used when interpreting the data generated from circulating miRNAs. Some other endogenous and exogenous reference controls should be tested in future studies to further validate the normalization strategy.

The lower levels of miR-21 and miR-146 found in the current study could actually be an early molecular change associated with the disease since the majority of the studied samples were from newly diagnosed individuals. MicroRNA can be dynamic and will be affected by the stage of disease, inflammatory activity, tissue composition and activation of immune cells. The observed downregulation may thus reflect an initial molecular response instead of expression pattern in the established disease. Besides, decreased levels of miR-21 and miR-146 might reflect negative feedback regulation failure as they are important regulators of inflammatory signaling pathways including NF-κB-mediated responses during the early inflammatory stage. The greater decrease of tissue indicates local mucosal dysregulation, especially concerning miR-146. Differences between the present findings and previous reports may be attributed to variations in disease duration, activity, treatment status, sample type, and study population characteristics.

## 5 Conclusions

The study reveals a correlation between the deregulation of miR-21 and miR-146 in ulcerative colitis patients, indicating their potential role in inflammatory pathways associated with the disease. The observed downregulation of miR-21 and the dysregulated expression of miR-146 highlights their specific but interrelated roles in regulating important inflammatory pathways, such as the NF-κB pathway and immune responses mediated by cytokines.

It is possible that the miR-21 expression changes are linked to persistent intestinal inflammation and fail to heal mucosal defects as described in other studies. However, causal relationships were not attempted in the present study. By contrast, the downregulation of miR-146 found in this study might be a reflection of changes in the regulation of pathways involved in the inflammatory response in UC. Additional functional studies are needed to determine the biological implications of this observation.

All of the above results suggest that deregulation of miR-21 and miR-146 expression is linked to ulcerative colitis. These miRNAs could be interesting biomarker candidates; but for further studies on their role in disease pathogenesis or for therapeutic use, larger scale and multicenter studies and functional investigations are needed.

## CRediT authorship contribution statement

**Bilal Rmaidh Mohammed:** Conceptualization, Investigation, Methodology, Formal analysis, Data curation, Writing – original draft. **Huda Rafaa Sabbar AL-alwani:** Supervision, Project administration, Methodology, Data curation, Writing – review & editing. **Raghad Jawad Hussein A.L. Akayshee:** Investigation, Resources, Data curation.

## Declaration of competing interest

The authors declare that they have no known competing financial interests or personal relationships that could have appeared to influence the work reported in this paper.
